# Supplementary material for: Profiling of Burkholderia pseudomallei variants derived from Queensland’s clinical isolates
Source: Microbiol Spectr. 2025 Dec 30;14(2):e02437-25. doi: 10.1128/spectrum.02437-25 (PMC12889130; doi:10.1128/spectrum.02437-25)
Supplement: Supplemental figures — Fig. S1 and S2. [file spectrum.02437-25-s0001.docx]

Profiling of *Burkholderia* *pseudomallei* variants derived from Queensland’s clinical isolates

Pauline M.L. Coulon^1^, Kay Ramsay^3^, Aven Lee^2^, Edita Ritmejeryte^2^, Miranda E. Pitt^1^, Joyce To^1^, Daniel G. Mediati^1^, Ian Gassiep^3^, Sarah Reed^2^, Patrick N. A. Harris^3^ and Garry S.A. Myers^1^

Affiliations:

^1^Australian Institute for Microbiology and Infection, Faculty of Science, University of Technology Sydney, NSW, Australia

^2^ Mass Spectrometry Facility, University of Queensland, Centre for Clinical Research, Queensland, Australia

^3^The University of Queensland Centre for Clinical Research (UQCCR), Faculty of Medicine, The University of Queensland, Queensland, Australia

Corresponding author:

Pauline M.L. Coulon, Australian Institute for Microbiology and Infection, Faculty of Science, University of Technology Sydney, NSW, Australia, +61390353555, [pauline.coulon@uts.edu.au](mailto:pauline.coulon@uts.edu.au)

**Supplementary Figures**

**Fig S1 Predicted structure of proteins carrying genomic mutations in TSV82 smooth compared to rough colony.** A) MbaJ B) PotF C) TSV82_RS_18625 D) TSV82_RS_19610.

**Fig S2 Predicted secondary structures of candidate regulatory ncRNA found within regions of genomic variability.** A) Contig_1 2552774 Bp TSV82 B) Contig_2 538080 Bp TSV82 C) Contig_2 2200644 Bp TSV82 D) Contig_2 2170684 Bp TSV287. Scale bar represents the individual base-paired probability of the RNA transcript.
